# Protein characterization of intracellular target-sorted, formalin-fixed cell subpopulations

**DOI:** 10.1038/srep33999

**Published:** 2016-09-26

**Authors:** Jessica S. Sadick, Molly E. Boutin, Diane Hoffman-Kim, Eric M. Darling

**Affiliations:** 1 Department of Molecular Pharmacology, Physiology, and Biotechnology, Brown University, Providence, RI, USA; 2 Center for Biomedical Engineering, Brown University, Providence, RI, USA; 3 Brown Institute for Brain Science, Brown University, Providence, RI, USA; 4 School of Engineering, Brown University, Providence, RI, USA; 5 Department of Orthopaedics, Brown University, Providence, RI, USA

## Abstract

Cellular heterogeneity is inherent in most human tissues, making the investigation of specific cell types challenging. Here, we describe a novel, fixation/intracellular target-based sorting and protein extraction method to provide accurate protein characterization for cell subpopulations. Validation and feasibility tests were conducted using homogeneous, neural cell lines and heterogeneous, rat brain cells, respectively. Intracellular proteins of interest were labeled with fluorescent antibodies for fluorescence-activated cell sorting. Reproducible protein extraction from fresh and fixed samples required lysis buffer with high concentrations of Tris-HCl and sodium dodecyl sulfate as well as exposure to high heat. No deterioration in protein amount or quality was observed for fixed, sorted samples. For the feasibility experiment, a primary rat subpopulation of neuronal cells was selected for based on high, intracellular β-III tubulin signal. These cells showed distinct protein expression differences from the unsorted population for specific (phosphorylated tau) and non-specific (total tau) protein targets. Our approach allows for determining more accurate protein profiles directly from cell types of interest and provides a platform technology in which any cell subpopulation can be biochemically investigated.

The brain is a complex organ comprised of physically intertwining and chemically interdependent cell populations. Accurately characterizing brain cell subpopulations is a necessary step for understanding normal and pathological neurobiology, as individual cell types may be disparately affected by stimuli, environmental conditions, or disease states^1^,^2^. However, identifying specific molecular properties, as well as differences in ubiquitously expressed proteins, for cell subpopulations poses a significant methodological challenge. Traditional identification of nervous system cells has been reliant on morphology, anatomical location, electrophysiology, immunohistochemical markers, retrograde tracers, and/or generation of transgenic models^2^,^3^,^4^,^5^. Commonly, for characterization studies, a region of the brain is isolated, cultured, and analyzed^3^,^6^. By processing heterogeneous samples without initial purification or enrichment, the expression levels of sparse subpopulations may become masked in the average, particularly if the protein(s) of interest (POI) is not unique to the subpopulation cell type. Subsequent genomic or proteomic testing of these mixed-population samples are biased by the large percentage of non-target cell types as well as by the non-physiological conditions attributed to *in vitro* culturing^2^,^7^. To effectively assess cell subpopulations, samples can be directly isolated from tissues, enriched specifically for the subpopulation, and analyzed to establish more accurate protein expression profiles.

Many techniques commonly used to study subpopulations are hindered by limited yields or throughput, inability to perform quantitative assays (e.g., immunohistochemistry), highly technical and time-consuming procedures (e.g., laser capture microdissection), or require genetic modification or low-efficiency transfection (e.g., lineage tracing, GFP-fusions)^8^,^9^. Single-cell analyses are ideal for analyzing cell-to-cell variability, but these techniques are prone to false negatives and may be less reproducible than data gathered from pooled cells^3^,^6^. Fluorescence-activated cell sorting (FACS) overcomes some of these limitations by rapidly separating large numbers of cells based on size, granularity, and molecular phenotype with minimal non-target cell contamination^3^. Specific POIs may be fluorescently tagged using retrograde tracers^10^, generating transgenic mouse lines^5^,^11^,^12^,^13^, or labeling cell surface markers^14^,^15^,^16^. While these methods are appropriate for certain studies, they limit researchers to using transgenic-modified, non-human species or a small subset of membrane-associated, targeting proteins with variable specificity for a given cell type. To improve upon these methodologies, we prepared samples for FACS by fluorescently labeling intracellular proteins that are characteristic of the target cell type. By doing so, subpopulations can be targeted more specifically with a broad array of available antibodies. Previous groups have shown the feasibility of this approach^17^,^18^, but none have subsequently analyzed the resulting subpopulations for characteristic protein expression.

Successful sorting of samples based on intracellular markers requires fixation, which can be problematic for downstream assays that rely on the separation of proteins for detection. In our method, we used 10% buffered formalin phosphate (10% formalin) because it is a highly common, cost-effective, and efficient fixative^19^. While not widely adopted beyond histology/cancer biology fields, extraction of proteins from formalin-fixed samples is an established technique, whereby formalin-fixed paraffin-embedded (FFPE) tissues are sectioned and subjected to high heat and denaturing agents to de-crosslink formalin-protein bonds^20^,^21^,^22^,^23^,^24^. To our knowledge, no one has applied this technique to establish protein profiles of cell populations sorted by FACS.

In this study, we developed a novel, fixation/sorting/protein extraction method to determine more accurate protein expression in cell subpopulations. Our overall protocol involved the following steps: (1) cell isolation; (2) fixation; (3) immunolabeling for our target protein of choice, β-III tubulin (TUBB3), a common neuron-specific, intracellular marker^25^, followed by FACS; and (4) protein extraction from cell subpopulations for western blot (WB) analysis. Individual steps of the process were validated using neural lineage-specific cell lines. To evaluate feasibility in a biologically complex system, we applied our methodology to a heterogeneous mix of primary, neonatal rat brain cells in which subpopulations were identified by high and low TUBB3 signal, sorted, processed, and analyzed for disease-relevant, tau protein variant expression.

## Results

### Comparison of extracted proteins from fresh and formalin-fixed samples

**Figure 1:**
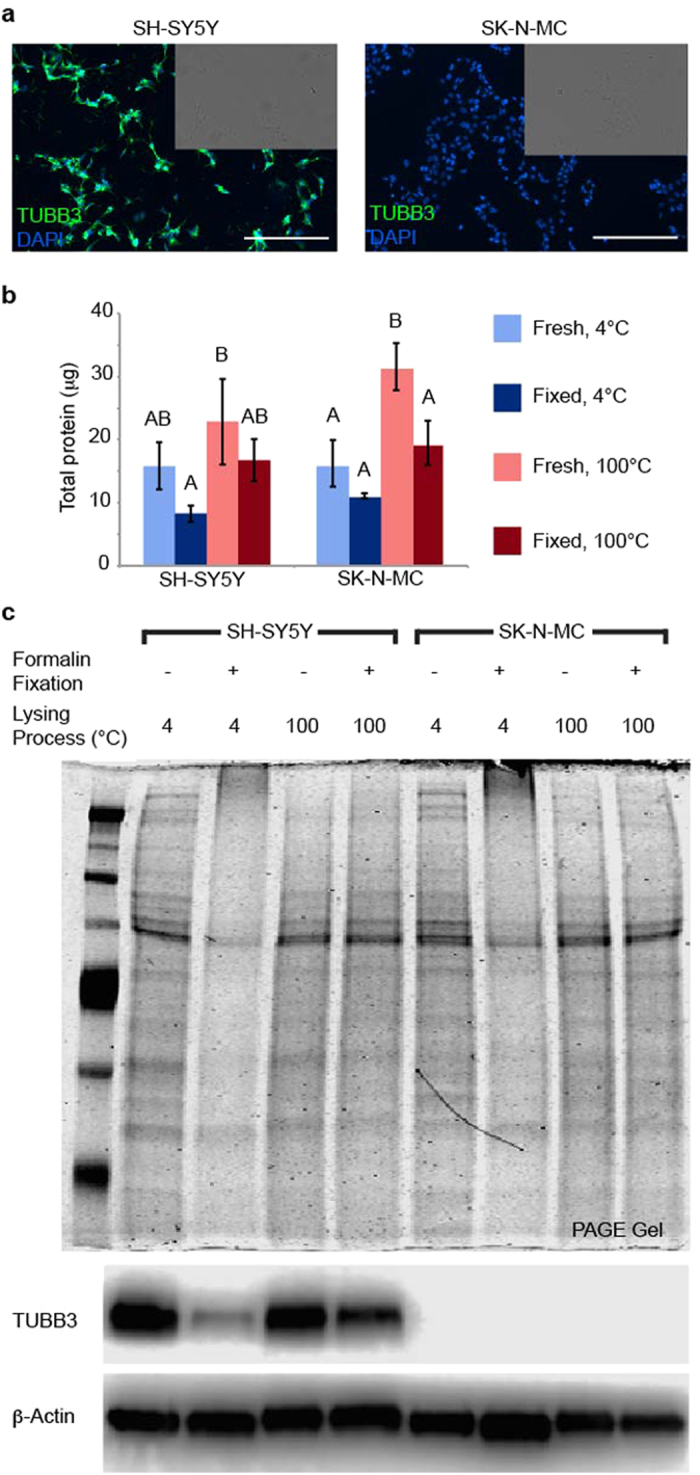
Protein extraction from formalin-fixed cells is comparable to fresh cells when lysing protocol includes high concentrations of Tris-HCl, anionic detergent SDS, and high heat. (**a**) Expression of TUBB3 (*green*) in cell lines SH-SY5Y and SK-N-MC. Scale bar, 200 μm. DAPI, 4′,6-diamino-2-phenylindole (*blue*). (**b**) Total protein isolated from formalin-fixed cells was compared to fresh cells under two different lysing conditions (4 °C, ice; 100 °C, boil). Data represented as mean ± standard deviation (s.d.) from four, independent experiments. Within cell lines, one-way ANOVAs were performed to determine significance (unlike letters are significantly different from each other, P < 0.02). (**c**) Protein quality assessed by Coomassie Blue stained PAGE gel for general banding and WB for POI.

A consistently reliable protocol for processing fresh and fixed samples was selected based on total protein isolation. To maintain a controlled biological system while validating protein extraction protocols, two cell lines were chosen based on their expression of the neuronal marker, TUBB3: SH-SY5Y (TUBB3+, “neuronal”) and SK-N-MC (TUBB3−, “non-neuronal”). As verified by immunofluorescence (IF), SH-SY5Y cells highly expressed TUBB3, while SK-N-MC cells had very low TUBB3 expression (Fig. 1a). Three different, commonly used protein extraction protocols, as inspired from FFPE literature, were evaluated based on Coomassie Blue stained general protein banding and WB^24^,^26^,^27^. Results showed that a lysis buffer containing 300 mM Tris-HCl, 2% sodium dodecyl sulfate (SDS), and 2X protease and phosphatase inhibitor cocktail^26^ assured maximal protein extraction from fixed samples without significant protein aggregation (Supplementary Fig. S1).

In addition to high Tris-HCl concentrations and the presence of the anionic detergent SDS, protein extraction of fixed samples required lysis by boiling (100 °C) to extract as much or more protein than that of fresh samples using the gold standard technique of lysis on ice (4 °C). Two protein extraction protocols, lysis on ice^27^ or boiling^24^,^26^,^28^, were evaluated based on total protein extracted as well as the previously described criteria. Total protein extracted was compared within cell lines (Fig. 1b). Within SH-SY5Y cell lysates, fresh samples lysed by boiling had three-times higher protein extraction yields than formalin-fixed samples on ice (P < 0.02). All other SH-SY5Y cell conditions were not significantly different from each other. Within SK-N-MC cell lysates, fresh samples lysed by boiling had protein extraction yields that ranged from 1.5–3 times higher than fresh samples lysed on ice, formalin-fixed samples lysed on ice, and formalin-fixed samples lysed by boiling (P < 0.01). All other SK-N-MC cell conditions were not significantly different from each other. As before, the presence of extensive protein aggregation was evaluated by Coomassie Blue staining, with results showing similar banding patterns across the entire length of polyacrylamide gel electrophoresis (PAGE) gels in all conditions except those fixed with 10% formalin and lysed on ice (lanes 3 and 7). In these lanes, protein clumps were found at the top of PAGE gels (>260 kDa). Subsequent WBs were probed for our POI, TUBB3 (Fig. 1c). In SH-SY5Y cell lysates, similar levels of TUBB3 expression were detected across fresh samples lysed on ice, fresh samples lysed by boiling, and fixed samples lysed by boiling. Meanwhile, lower TUBB3 levels were found in fixed samples lysed on ice. Trends in protein banding and TUBB3 expression were consistent over four independent runs (Supplementary Fig. S2). In all following experiments, protein from formalin-fixed samples was extracted using the boiling method.

### Intracellular target-based FACS protocol validation

**Figure 2:**
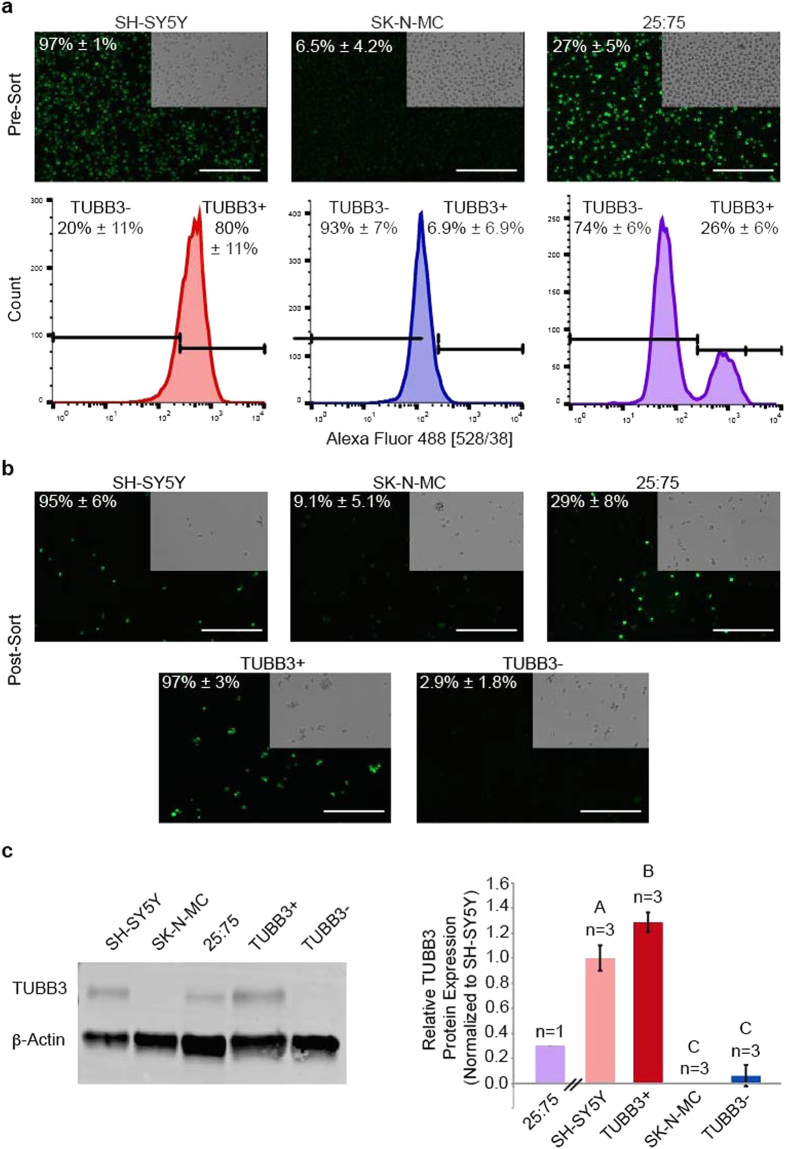
Cell line controlled ratio sorts and subsequent WBs support validity of method to enrich for cell subpopulations. (**a**) Representative cell populations pre-FACS and histograms collected during FACS. Ab-labeled, TUBB3+ (*green*) population quantified. (**b**) Representative images of post-FACS, collected cell populations. (**c**) Representative WB of post-FACS, collected cell populations for TUBB3 expression. Densitometry-based quantification of TUBB3-positive expression from three, independent experiments (mean ± s.d.). One-way ANOVA was performed to determine significance among all control and enrichment groups (unlike letters are significantly different from each other, P < 0.04). Scale bars, 200 μm.

**Figure 3:**
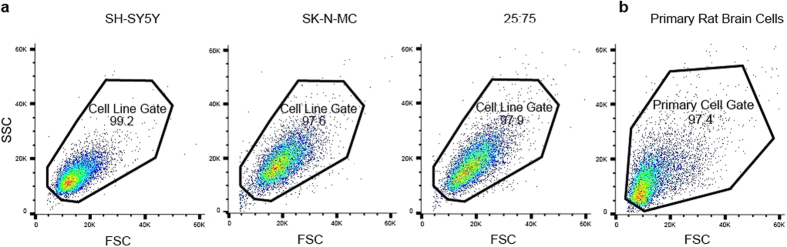
Forward vs. side scatter plots for (**a**) all cell line and (**b**) primary cell groups. Dots indicate sorted events included within the gated region. FSC, forward scatter; SSC, side scatter.

The ability to sort cell populations based on our intracellular target TUBB3 was evaluated by pre- and post-FACS fluorescence imaging and WB. In addition to TUBB3+ (SH-SH5Y cells only) and TUBB3− (SK-N-MC cells only) controls, cell lines were mixed together in a ratio of 25% SH-SY5Y cells: 75% SK-N-MC cells (25:75) to artificially establish a well-defined, heterogeneous population. As expected, imaging showed SH-SY5Y cells were primarily TUBB3+, SK-N-MC cells were primarily TUBB3−, and 25:75 cells were 27% TUBB3+ (Fig. 2a). All cell groups exhibited limited debris when assessing their forward and side scatter plots (Fig. 3a). Post-FACS, TUBB3 expression for cell groups remained consistent for all iterations of the experiment. SH-SY5Y cells had a single, primarily positive peak; SK-N-MC cells had a single, primarily negative peak; and 25:75 cells had two distinct peaks in which the positive peak (TUBB3+) had approximately one quarter of total events collected, indicating the cell separation worked as anticipated (Fig. 2a). Based on flow cytometric analysis, SH-SY5Y cells had a 20 ± 11% false negative rate, and SK-N-MC cells had a 7 ± 7% false positive rate. 25:75 samples had lower false positive and false negative rates (~3%) than those of single cell type samples. After sorting, fluorescence imaging of collected cells further corroborated these findings, showing that SH-SY5Y cells and TUBB3+ enrichment groups were primarily TUBB3+; both SK-N-MC cells and TUBB3− enrichment groups were primarily TUBB3−; and 25:75 unsorted group was 29% TUBB3+ (Fig. 2b).

To confirm the expected TUBB3 expression, post-FACS cells corresponding to positive (SH-SY5Y), negative (SK-N-MC), and mixed (25:75, +/−/unsorted) samples were assessed by WB (Fig. 2c). Previous validation experiments indicated that antibody labeling for FACS did not affect subsequent probing for the same antigen after processing samples for WB (Supplementary Fig. S3). Results showed that TUBB3 expression in the 25:75 unsorted sample was slightly higher than expected (~30% of the positive control). TUBB3+ and TUBB3− enriched groups had similar protein expression levels to the positive and negative samples, respectively, indicating the sort and protein assessment were successful. All cell line control and enrichment groups were statistically significant from each other (P < 0.04), except between SK-N-MC cells and TUBB3− enrichment groups. Trends in sorts and subsequent TUBB3 protein expression were consistent over three independent iterations (Supplementary Fig. S4).

### Neonatal rat brain cell feasibility experiment

**Figure 4:**
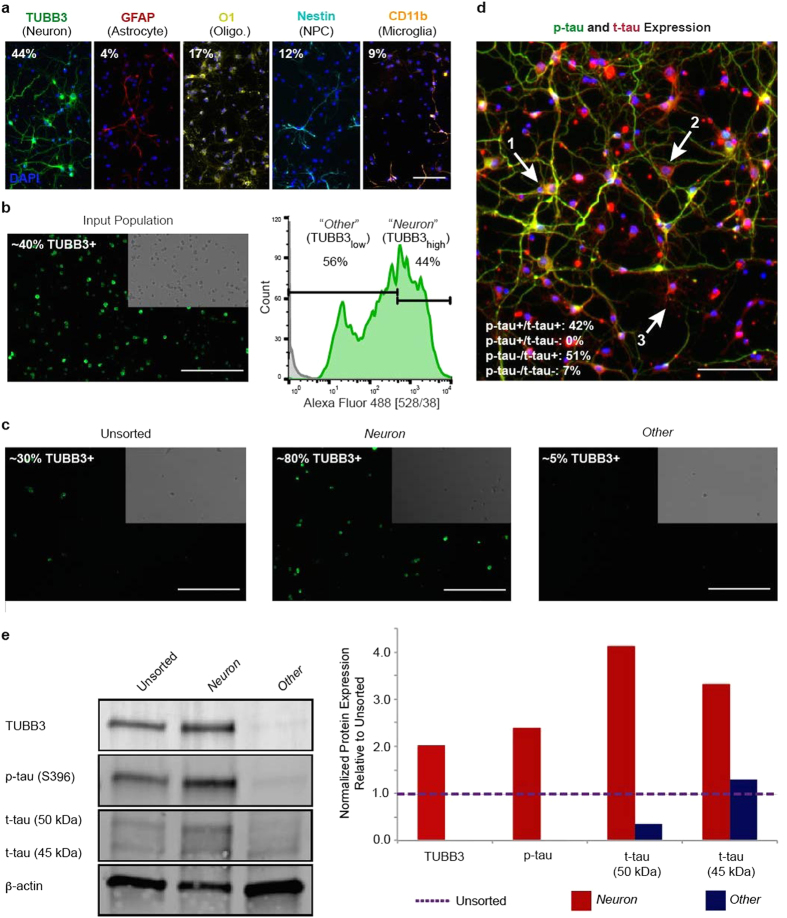
Heterogeneity found in neonatal rat brain cells underlines importance of subpopulation identification and enrichment for more useful/accurate protein characterization. (**a**) Representative images of primary, neonatal, rat brain cells cultured in 2D. Cell nuclei are labeled with DAPI (*blue*). Scale bar, 100 μm. (**b**) Representative image of TUBB3-immunostained, unsorted cells in suspension prior to enrichment and subsequent distribution of TUBB3-associated fluorescence levels (*green)*. Scale bar, 200 μm. (**c**) Representative images of post-FACS, collected cells. TUBB3 (*green*) population was quantified. Scale bars, 200 μm. (**d**) IF images of primary neonatal rat brain cells cultured in 2D. P-tau (*green*), t-tau (*red*), and DAPI (*blue*) populations were quantified. Identification of cell types was based on tau expression and morphology. (1) Neuron; (2) Astrocyte; (3) Oligodendrocyte. Scale bar, 100 μm. (**e**) WB and quantification of post-FACS, collected cells.

Neonatal rat brain cells were used as a representative model of cellular heterogeneity found in primary tissues. To visualize the variety of cell types present in these primary samples, cells were cultured on poly-D-lysine (PDL)-coated plastic for 4 days, and immunostained for standard, brain cell type markers. Specifically, neurons (TUBB3+), astrocytes (glial fibrillary acidic protein; GFAP+), oligodendrocytes (oligodendrocyte marker 1; O1+), neural progenitor cells (NPC) (nestin+), and microglia (CD11b+) were identified (Fig. 4a). Fluorescence image analysis showed that approximately 44% of 2D cultured cells were TUBB3+. Quantification of the other brain markers showed 4% GFAP+, 12% Nestin+, 9% CD11b+, and 17% O1+ for a single, independent run. The extent of co-expression for specific markers in these cells was not evaluated.

Subsequent to the cell line validation experiments, primary brain cells were pooled from nine rats, labeled with TUBB3, and sorted. The forward and side scatter plot for FACS showed limited debris along with less uniformity in size and granularity as compared to cell line runs (Fig. 3b). This heterogeneity was also observed for TUBB3 expression in brain cell isolates evaluated by fluorescence microscopy and FACS (Fig. 4b). Rather than a binary, positive-negative signal, multiple positive peaks were present. Due to the heterogeneous nature of TUBB3+ cells, the sample was split into TUBB3-high expressers (TUBB3_high_) in which the majority of TUBB3+ cells representing a “*Neuron*” population were captured and TUBB3-low expressers (TUBB3_low_) in which all “*Other*” cells were captured. While TUBB3 is not a completely exclusive marker for neurons, it was used as such for demonstration purposes in this study. The gating threshold took into account both TUBB3 signal level – above the third decade of fluorescence as established from cell line sorts – and cell numbers, which had to be sufficiently high to allow for subsequent protein analysis. In total, 44% of cells were collected in the *Neuron* group, and 56% of cells were collected in the *Other* group. Unsorted and sorted cells were imaged post-FACS. The unsorted group was composed of ~30% TUBB3+ cells; the *Neuron* group was ~80% TUBB3+ cells; and the *Other* group was ~5% TUBB3+ cells (Fig. 4c). Sorting success was verified by WB (Fig. 4e). As compared to the unsorted group, TUBB3 expression was 100% higher in the *Neuron* group and negligible in the *Other* group.

Beyond TUBB3 expression, samples were probed for other POIs. Specifically, two tau proteins were chosen to highlight the usefulness of this method to study 1) proteins specific to a single cell type and 2) expression levels of ubiquitous proteins in a single cell type. Paired helical filament-1-specific, phosphorylated tau (p-tau) binds to a single phosphorylated residue (at serine 396, for respective antibody used) and was chosen because, in neonatal rats, it is only present in neurons^29^,^30^. Total tau (t-tau) binds to all six isoforms of tau and was chosen because it is found at different expression levels in neurons, astrocytes, and oligodendrocytes^31^,^32^. Both proteins were detected by IF in heterogeneous brain cell, 2D cultures for a single feasibility experiment (Fig. 4d). WB assessment of these tau isoforms in sorted groups showed the feasibility of examining protein expression in a given cell type without bias from the larger population. In the *Neuron* group, p-tau expression increased by 150%, and t-tau expression increased 200–300% (for molecular weight bands, 45 and 50 kDa, respectively) relative to the unsorted sample (Fig. 4e). In the *Other* group, p-tau expression was negligible, and t-tau expression was overall lower as compared to the unsorted sample.

## Discussion

In the current study, we established and validated a novel fixation/sorting/protein extraction method in which cell subpopulations can be identified and collected based on an intracellular marker and their protein expression characterized without bias from the larger population. By fixing cells immediately after tissue dissociation, representative cellular heterogeneity was maintained, and respective molecular expression was preserved^22^,^33^. This process allowed maintenance of cell characteristics outside original physiological environments without invoking potential changes due to standard 2D cell culturing conditions^7^,^19^. Furthermore, by fixing samples, cells could be immunolabeled and subsequently sorted based on intracellular markers, which provides investigators with vastly more cell type-specific targets^18^. By taking advantage of commercially available antibodies, this protocol can theoretically be applied to any cell or animal model of interest.

While fixation and sorting based on intracellular markers has been used previously to obtain cell subpopulations, these studies limited analyses to gene expression^17^,^18^. Commonly, mRNA abundance has been used as a proxy for protein abundance, which assumes that mRNA presence is the major factor determining the amount of protein made^34^. However, overall mRNA abundance has a relatively low correlation with protein synthesis (squared Pearson’s coefficient of ~0.4) due to post-transcriptional modification and regulation^35^,^36^. Therefore, protein characterization provides the most concrete representation of cell phenotype^36^. Through our method, we employed a high throughput, user-friendly procedure in which common and commercially available materials were used to study protein expression in specified cell populations. Collected cell lysates were used for the study of many different proteins, underlining this protocol’s usefulness in establishing more accurate protein profiles for specific cell types.

Lysis buffer composition and high heat were both critical for successful isolation of proteins from fixed samples. The chosen lysis buffer was made of a high concentration of Tris-HCl and 2% SDS. Tris-HCl has been implicated in helping the formalin fixation reversal process by acting as a formaldehyde scavenger and/or may direct formaldehyde crosslinks through transamination^26^. SDS both denatures and acts as a detergent on fixed proteins^22^. Lastly, by heating samples to boiling temperatures, proteins become less tangled, and methylene bridges may undergo partial thermal hydrolysis^21^,^22^. Of note, regardless of cell type, fresh samples lysed by boiling had significantly higher total protein collected. Based on these findings, denaturing of proteins due to the exposure of high heat and SDS may increase availability of proteins to be collected as compared to standard WB lysing conditions in which samples remain on ice. However, this processing is not suitable for all protein assays, particularly those that depend on maintenance of physiological structure (e.g., co-immunoprecipitation, chromatin immunoprecipitation, two-hybrid system, x-ray crystallography).

Intracellular target-based FACS validation experiments worked as anticipated, as demonstrated using pure or proportioned populations of neural cell lines. TUBB3 expression was largely consistent between cell line controls and respective, positive/negative enrichment groups. Interestingly, the enriched TUBB3+ group exhibited higher TUBB3 expression by WB densitometry than the pure, positive control group (SH-SY5Y cell line). This result was driven by the variability in TUBB3 expression levels that exist even in SH-SY5Y cells, which by flow cytometry showed only 80% positive cells (Fig. 2a histograms). Overall, TUBB3 expression between positive conditions (SH-SY5Y and TUBB3+) and negative conditions (SK-N-MC and TUBB3−) was clearly distinct, even when accounting for possible false positive/negative signals.

Brain cells from rat neonates were used as a representative model of cellular heterogeneity found in primary tissues. While TUBB3 is a standard neuronal marker, expression level of this protein can vary significantly across neural cell types^37^,^38^. The current study supported this observation, with flow cytometric data for TUBB3 expression in neonatal rat brain cells ranging over multiple decades of fluorescence. The feasibility portion of this study focused on separating and analyzing protein expression in neurons; however, many other cell types of potential interest were also present in the harvested brain cell isolate. Astrocytes, oligodendrocytes, microglia, and NPCs each comprised small portions of the total cell population. The presented method could be applied to any of these cell types to evaluate unique or ubiquitous protein characterization without bias from the larger population. While exact cell composition for complex, developing organs like the brain may vary with animal model and age, the need for robust enrichment strategies that allow for characterizing protein expression in important cell types is clear.

The importance of our demonstrated method lies in its ability to assess multiple POIs, which may be cell type-specific or ubiquitously expressed, in isolated cell subpopulations. As a feasibility experiment, two tau proteins were chosen to illustrate this point. Tau is a major microtubule-associated protein that helps maintain the stability of axonal and cellular processes by allowing microtubules to interact with other cytoskeletal elements, such as spectrin and actin filaments^29^,^39^,^40^. In healthy brains, tau is primarily found in neurons, but non-neuronal cells, such as astrocytes and oligodendrocytes, also have low expression of tau^31^,^32^,^41^,^42^,^43^. Due to alternative splicing on the *MAPT* gene, there are six different tau isoforms, which are differentially expressed throughout development and may not be equally expressed across all types of neurons^39^,^41^,^42^. In fetal and neonatal neurons, p-tau (at serine 396) is highly abundant, which may be due to the dynamic role p-tau plays in establishing neuronal architecture^30^,^42^. This was confirmed for isolated, primary, neonatal rat brain cells, as detected by both IF and WB. The *Neuron* enrichment group, in which cells had the highest expression of TUBB3, were co-expressive for p-tau, as was previously established by Tashiro and colleagues^29^. In contrast, t-tau (in which all tau isoforms are recognized) was detectable in both *Neuron* and *Other* enrichment groups, although at different levels with t-tau being higher in *Neuron* and lower in *Other* cell populations. By selectively isolating high and low TUBB3-expressing cells, proteins like tau may be studied in specific cell populations without being biased by other resident cell types. This may be especially advantageous when considering the role of tau and the development of neurodegenerative diseases. Under pathological conditions, including Alzheimer’s disease, Pick’s disease, and ischemic stroke, variations in abnormal tau expression and tau aggregate formation are found in different anatomical regions, cell types, cell type subpopulations, isoform ratios, and intensities of phosphorylation^32^,^39^,^41^. Classically, characterization of tau pathology has been limited to immunohistochemistry, electron microscopy, standard immunoblotting, and identification of morphological features^41^. Due to the inherent variation in tau, the described method may clarify the role of cell subpopulations in disease progression and potential identification of therapeutic strategies^44^,^45^.

The limitations of this method align primarily with those inherent in the individual procedures and analysis techniques. The cell subpopulation being enriched has to be identifiable based on a specific, molecular marker, which may not be known for all cell types of interest. Antibody specificity, cell preparation, and POI localization can all contribute to loss of signal^17^,^46^,^47^. Large cell numbers, relative to the rarity of the cell type being examined, are required to account for cell loss during FACS and to assure sufficient cell yield needed for successful protein extraction; however, this may be remedied by using more sensitive protein assays, such as enzyme-linked immunosorbent assays (ELISAs)^48^. Also, FACS sample gating may disproportionately exclude certain cell types. This was evident in the feasibility experiment, where TUBB3 expression in the unsorted population changed from ~40% (pre-FACS) to ~30% (post-FACS), indicating that a disproportionate number of TUBB3+ cells fell outside the FSC-SSC gate. The method is not applicable to live cells, which obviates the possibility for functional assays, but does provide a novel means for studying protein expression in sparse subpopulations. Our major goals were to obtain more accurate protein profiles directly from cell types of interest and to establish a platform technology in which any cell subpopulation can be biochemically investigated using common, downstream analyses.

Our presented method provides a platform from which cell subpopulation protein characterization can be investigated for both *cell type-specific* and *ubiquitously expressed* proteins. Combinations of intracellular markers allow for the collection of more specific cell subpopulations than extracellular, surface markers alone, while still allowing for protein assessment using quantitative assays such as WB and, possibly, ELISA and proteomics. While TUBB3 expression in the brain was demonstrated as a feasible target, this platform methodology is compatible with virtually any fluorescent antibody and can be applied to study many other relevant cell sources and tissues made complicated by heterogeneity.

## Materials and Methods

### Antibodies

Primary antibodies used in the study included: Mouse anti-TUBB3 (801201, 1:500 for WBs and IF, Biolegend), rabbit anti-GFAP (Z0334, 1:1000 for IF, Dako), mouse anti-nestin (MAB353, 1:200 for IF, Millipore), mouse anti-CD11b (CBL1512, 1:50 for IF, Millipore), mouse anti-O1 (MAB344, 1:50 for IF, Millipore), rabbit anti-TUBB3 (ab18207, 1:100 for sorts, Abcam), mouse anti-β-actin (ab6276, 1:1,000 for WB, Abcam), mouse anti-TAU-5 (refers to t-tau; ab80579, 1:500 for WB and 1:100 for IF, Abcam), and rabbit anti-tau (phospho S396) (ab109390, 1:1,000 for WB and 1:100 for IF, Abcam). For sorting studies, primary antibodies were detected with goat anti-rabbit IgG (H&L) secondary antibody conjugated to Alexa Fluor 488 (A11008, 1:100, Life Technologies). For IF studies, primary antibodies were detected with either goat anti-rabbit IgG (H&L) Alexa Fluor 488 (A11008, 1:500, Life Technologies) or Cy5 AffiniPure goat anti-mouse IgG (H&L) (115-175-146, 1:500, Jackson ImmunoResearch). For WB using chemiluminescence detection, primary antibodies were detected using rat anti-mouse IgG1 HRP-conjugated secondary antibody (18-4015-82, 1:10,000, Affymetrix eBioscience) or goat anti-mouse IgG2a HRP-conjugated secondary antibody (sc-2061, 1:5,000, Santa Cruz Biotechnology). For WB using near-infrared (NIR) detection, primary antibodies were detected using IRDye 800CW goat anti-mouse (925–332210) or IRDye 680RD donkey anti-rabbit (925–68073) secondary antibodies (1:15,000, LI-COR). All antibodies were diluted in 1–5% wt/vol (1% for IF; 3% for sorts; 5% for WBs) bovine serum albumin (BSA; Fisher Scientific) (Fraction IV) in phosphate buffered saline (PBS) or 1X tris-buffered saline tween (TBST).

### Cell lines and reagents

Two different cell lines were used in this study: SH-SY5Y (human, bone marrow-derived neuroblastoma) and SK-N-MC (human, brain-derived neuroepithelioma). SH-SY5Y cells (a gift from Dr. Eric Morrow, purchased from ATCC, CRL-2266, lot# 59121171) were expanded in high glucose DMEM (HyClone, GE Healthcare) with 10% FBS (Zen-Bio, lot# 08152003), 1% penicillin/streptomycin (HyClone, GE Healthcare), and 1% Gluta-Max (HyClone, Thermo Scientific). SK-N-MC cells (purchased from ATCC, HTB-10, lot# 58078653) were grown in MEM (HyClone, GE Healthcare) supplemented with 10% FBS and 1% penicillin/streptomycin. Cultures were maintained in incubators at 37 °C with 5% CO_2_ and were passaged when cell confluence reached 60–80% using 0.25% trypsin-EDTA (HyClone, GE Healthcare). Cell lines were recently authenticated using short tandem repeat profiling by ATCC, which confirmed that both cell lines were exact matches for corresponding ATCC standards.

### Primary cell isolation

**Figure 5:**
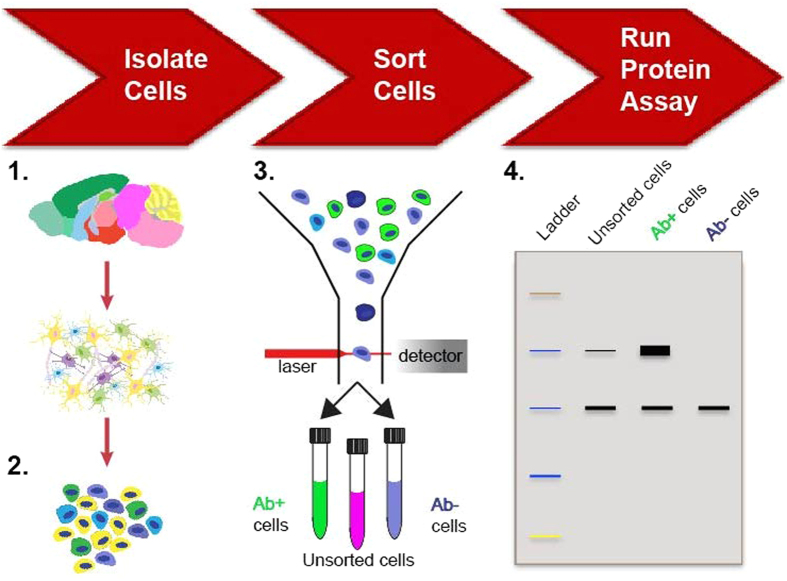
Methodology schematic. (1) Isolate tissue of interest and dissociate cells; (2) Fix samples in suspension with 10% formalin; (3) Label cells with a specific intracellular marker for the cell population of interest and select for cells using FACS; (4) Extract protein from collected samples, and run protein assays. Ab, antibody.

Brown University’s Institutional Animal Care and Use Committee approved all protocols related to primary cell isolation (IACUC #1409000090A002), and the methods were carried out in accordance with the approved protocols. Whole brains were isolated from postnatal day 1–2 CD rats (Charles River). Cell isolation followed a modified BrainBits protocol^49^ (Fig. 5, Part 1). Briefly, meninges were removed from whole brains while submerged in Hibernate A buffer (BrainBits) supplemented with 1X B27 (Invitrogen) and 0.5 mM Gluta-Max. Each brain was minced into ~2 mm^3^ pieces and digested in 2 mg/mL papain (BrainBits) solution dissolved in Hibernate A without calcium buffer (BrainBits) at 30 °C for 30 minutes. After removing the papain solution, tissues were titurated 25 times with a fire polished Pasteur pipette in Hibernate A/B27 buffer. The cell suspension was centrifuged for 5 minutes at 150 × g and was resuspended in culture medium composed of Neurobasal A medium (Invitrogen) supplemented with 1X B27, 0.5 mM Gluta-Max, and 1X penicillin/streptomycin. To remove cellular debris, resuspended cells were passed through a 40 μm cell strainer, centrifuged for 5 minutes at 150 × g, and resuspended in culture medium. Cell straining, centrifugation, and resuspension were repeated once more. Cell viability and yields were calculated by using a trypan blue exclusion assay (HyClone, Thermo Scientific). On average, cell viability was 97% (±1%), and cell yields were 37 × 10^6^ (±3 × 10^6^) cells per pup.

### Immunofluorescence

Cell line cultures were seeded onto sterile 15 mm^2^ circular glass coverslips at 85,000 cells/cm^2^ and grown for 4 days. Primary, neonatal rat brain cells were seeded onto 50 μg/mL PDL (Sigma)-coated wells at a density of 72,000 cells/cm^2 ^and were also cultured for 4 days. Cell line cultures were fixed with 10% formalin (Fisher Scientific), while primary, neonatal rat cultures were fixed with 4% vol/vol paraformaldehyde (Electron Microscopy Science) supplemented with 8% wt/vol sucrose (Fisher Scientific). In both cases, cells were fixed for 10 minutes. Following fixation, samples were washed three times with 1X PBS (standard for all wash steps) and permeabilized with 0.1% vol/vol Triton X-100 (Fisher Scientific) in 1X PBS for 10 minutes. Cultures were washed and blocked with 3% wt/vol BSA in 1X PBS for 1 hour. Primary antibodies were incubated with samples for 1 hour at room temperature or overnight at 4 °C. After washing, secondary antibodies were incubated with samples for 1 hour at room temperature. Lastly, samples were washed, and nuclei were stained with 1 μg/mL DAPI (Thermo Fisher Scientific) in 1X PBS. Images were taken on a Cytation 3 Reader (BioTek Instruments) or a Nikon Eclipse Ti-U epifluorescent microscope (Nikon Instruments) with a QICAM digital camera (QImaging). Quantification was based on raw, untouched images using Gen5 Data Analysis Software to count nuclei as well as positively antibody-labeled cells (BioTek Instruments). Images shown were enhanced by adjusting brightness and contrast uniformly across the entire image to show morphology. All cell line and primary cell results were from single experiments with 3–4 technical replicates, respectively.

### FACS based on intracellular TUBB3 expression

#### Cell Line Sorts

To determine how well our proposed method resolved isolating cell subpopulations under idealized conditions, cell lines SH-SY5Y and SK-N-MC were used as positive and negative controls for TUBB3 expression, respectively, our chosen marker for subsequent sorting (Fig. 5, Part 3). Briefly, cell lines were uplifted using 0.25% trypsin-EDTA, centrifuged for 5 minutes at 400 × g, resuspended in their respective culture medium, and counted using the trypan blue exclusion assay. Cells were again centrifuged for 5 minutes at 400 × g and resuspended at 10 × 10^6 ^cells/mL in 10% formalin for 10 minutes. For each unsorted condition, 1–2 mL of the cell suspension were processed by FACS, and for each sorted condition, 3-6 mL of the cell suspension were processed. Cells were then centrifuged for 5 minutes at 2,500 × g and resuspended in 1X PBS. At this point, cells were split into four groups: (1) Cell line only: no antibody labeling control; (2) Cell line only: secondary antibody labeling control; (3) Cell line only with both primary and secondary antibody labeling; and (4) Mixed ratio of 25% SH-SY5Y cells: 75% SK-N-MC cells (25:75) with full antibody labeling. Volumes of each sample fit within 1.7 mL snap cap tubes. All seven samples were centrifuged for 5 minutes at 2,500 × g and washed three times with 1X PBS. Samples were resuspended at 10 × 10^6 ^cells/mL in 1X PBS and stored overnight at 4 °C. The following day, after cells were washed once with cold, 1% wt/vol BSA in 1X PBS (wash buffer), samples were incubated with 0.5% vol/vol Tween20 (Fisher Scientific) for 15 minutes in the dark at room temperature to permeabilize cells. Samples were washed once with cold wash buffer and then blocked with cold, 3% wt/vol BSA for 30 minutes at room temperature. Cells were incubated with primary antibodies for 30 minutes at room temperature, followed by two iterations of centrifugation (5 minutes at 2,500 × g) and washes with cold wash buffer. Then, cells were incubated with secondary antibody for 30 minutes at room temperature in the dark, followed by two iterations of centrifugation (5 minutes at 2,500 × g) and washing with cold wash buffer. Finally, a sample of cells from each group was counted, and each sample was resuspended at 20 × 10^6 ^cells/mL in 1X PBS.

Directly prior to sorting, cells were passed through a 40 μm filter. An Influx high-speed cell sorter (BD Bioscience) using a 488 nm laser and a 530/30 bandpass filter was used to sort cells. Non-antibody-labeled controls were used to gate forward and side scatter profiles for both cell lines. Secondary-only controls were used to establish fluorescence baseline noise. Fully antibody labeled SH-SY5Y cells were used to gate positive TUBB3 expression, and fully antibody labeled SK-N-MC cells were used to gate negative TUBB3 expression. Single cell line, fully antibody-labeled groups were run through the sorter and collected in their entirety (i.e., collected based on gating their forward and side scatter profiles and exclusion of any doublets or triplets). The 25:75 group was sorted into TUBB3+ and TUBB3− populations. All samples were collected into 50 mL falcon tubes, and a small sample of collected cells was imaged. All other cells were immediately prepared for protein extraction. Results were from three, independent experiments. All results from sorts were graphed using FlowJo 10.1 software.

#### Primary Cell Sort

To explore the feasibility of the proposed method for a heterogeneous, primary cell population, cells were isolated and pooled from the whole brains of 9 rat neonatal pups. After cell isolation and dissociation, cells were immediately fixed with 10% formalin in suspension for 10 minutes, washed three times with 1X PBS, and incubated overnight at 4 °C in 1% wt/vol BSA in 1X PBS to minimize cell clumping (Fig. 5, Part 2). Cells were then split into four groups: (1) No-antibody labeling control; (2) Secondary antibody-only control; (3) Fully antibody-labeled sample collected in its entirety through the FACS instrument (unsorted); and (4) Fully antibody-labeled sample sorted based on positive and negative TUBB3 expression. 90 × 10^6 ^cells (e.g., 9 mL cell suspension) were used for the unsorted condition, and 180 × 10^6 ^cells (e.g., 18 mL cell suspension) were used for the sorted condition. Antibody labeling followed the procedures described above. Since TUBB3 expression in primary cells spanned a three-decade continuum, gating was designated by fluorescence intensity for the upper third (TUBB3_high_; “*Neuron*” enrichment group) and lower two-thirds (TUBB3_low_; “*Other*” enrichment group). This gating corresponded to the fluorescence profile for positive and negative cell lines. Data were collected from a single feasibility run (n = 9 rats, cells pooled), and results were graphed using FlowJo 10.1 software.

### Total protein isolation and quantification

To investigate parameters required to extract protein from formalin-fixed cells, SH-SY5Y cells and SK-N-MC cells lines were used as representative samples, and three lysis buffers (varying in concentration of Tris and addition of SDS) were assessed based on their general protein banding profile and WB protein expression for our POI TUBB3. Ultimately, the most reliable lysis buffer was composed of 300 mM Tris-HCl (Fisher Scientific) at pH 8, with 2% SDS (Fisher Scientific) and 2X Halt Protease and Phosphatase Single-Use Inhibitor Cocktail (Pierce, Thermo Scientific)^26^. Two lysis protocols were evaluated: (A) 30 minutes on ice (standard for WB sample preparation^27^) or (B) 30 minutes at 100 °C followed by 2 hours at 60 °C (boiling method; accepted for FFPE samples^21^,^24^,^50^). All samples were then centrifuged at 4 °C for 10 minutes at 14,000 × g, and supernatants were transferred to fresh microcentrifuge tubes. Total protein concentration was determined using the BCA assay (Pierce, Thermo Scientific), and protein quality was assessed using Coomassie Blue (MP Biomedicals LLC) to stain general protein banding in equally loaded PAGE gels (Bio-Rad). Protein extracted from fresh samples lysed on ice was used as a positive control, while protein extracted from fixed samples lysed on ice was used as a negative control. In all subsequent cell line and primary cell sorts, collected samples were lysed using the boiling method (Fig. 5, Part 4). For the validation experiment using cell lines, between 15–150 μg (corresponds to estimated 5–30 × 10^5^ cells) protein were collected per sorted group, and for the feasibility experiment using primary cells, 15–18 μg (corresponding to estimated 1–2 × 10^5 ^cells) protein were collected per sorted group.

### Western blot

WBs were run to assess POI expression (Fig. 5, Part 4). To validate lysing protocols, WBs were used to compare POI expression between formalin-fixed and fresh cells. In sorting studies, POI expression was used to evaluate the success of FACS enrichment based on a specific, intracellular marker. Specifically, TUBB3 was used as the intracellular marker of interest, and in the feasibility experiment, *Neuron* and *Other* enriched populations were evaluated for other POIs, such as p-tau and t-tau.

Equal amounts of total protein (5 μg per lane; corresponding to estimated 2 × 10^4^–1 × 10^5^ cells) were separated on precast PAGE gels and transferred onto Immobilon IP (for chemiluminescence visualization) or Immobilon IF (for NIR visualization) polyvinylidene fluoride (PVDF) membranes (Millipore). Ponceau S (Acros Organics) stain was used to confirm successful protein transfer. Membranes were blocked with 5% BSA in 1X TBST (10 mM Tris-HCl (pH 7.6), 15 mM NaCl, 0.05% Tween 20) for chemiluminescence studies or 5% BSA in 1X PBS for NIR studies. Blots were incubated overnight with primary antibodies. Blots were washed three times in 1X TBST and incubated with secondary antibodies (HRP-conjugated for chemiluminescence or NIR-conjugated for NIR) for 1 hour at room temperature. For chemiluminescence studies, blots were developed using 5-Amino-2,3-dihydro-1,4-phthalazinedione (MP Biomedicals LLC) and p-courmaric acid (MP Biomedicals LLC) and were visualized on a C-Digit Blot Scanner (LI-COR). For NIR studies, blots were scanned on an Odyssey Infrared Imaging System (LI-COR) and were assessed by densitometry using ImageJ (NIH) software. NIR blots were stripped with NewBlot PVDF Stripping Buffer (LI-COR) and were reprobed up to three times to allow for detection of multiple POIs with similar molecular weights on the same blot.

WBs for the lysing protocol validation were run from 4 independent experiments, with 1-2 technical replicates per experiment. For cell line sorting experiments, WBs were run from 3 independent experiments, each with 2 technical replicates. WB results from the primary neonatal rat cell sort were from a single experiment with 2 technical replicates.

### Statistical Analysis

Sigma Plot version 12.5 (Systat Software Inc.) was used to perform all statistical analyses. All data sets passed Shapiro-Wilk normality and equal-variance tests. Data presented are displayed as arithmetic mean (±s.d., if applicable). One-way ANOVAs were used to detect significance for all experiments involving multiple comparisons (processing conditions, sorted cell groups). Significance levels were determined using Bonferroni post-hoc tests (P < 0.05).

## Additional Information

**How to cite this article**: Sadick, J. S. *et al*. Protein characterization of intracellular target-sorted, formalin-fixed cell subpopulations. *Sci. Rep.* **6**, 33999; doi: 10.1038/srep33999 (2016).

## Supplementary Material

Supplementary Information

## References

[b1] Underwood E. The brain’s identity crisis. Science 349, 575–577 (2015). 26250665 10.1126/science.349.6248.575

[b2] Fishell G. & Heintz N. The neuron identity problem: form meets function. Neuron 80, 602–612, doi: 10.1016/j.neuron.2013.10.035 (2013). 24183013

[b3] Okaty B. W., Sugino K. & Nelson S. B. Cell type-specific transcriptomics in the brain. J Neurosci 31, 6939–6943, doi: 10.1523/JNEUROSCI.0626-11.2011 (2011). 21562254 PMC3142746

[b4] Zeisel A. . Cell types in the mouse cortex and hippocampus revealed by single-cell RNA-seq. Science 347, 1138–1142 (2015). 25700174 10.1126/science.aaa1934

[b5] Lobo M. K., Karsten S. L., Gray M., Geschwind D. H. & Yang X. W. FACS-array profiling of striatal projection neuron subtypes in juvenile and adult mouse brains. Nat Neurosci 9, 443–452, doi: 10.1038/nn1654 (2006). 16491081

[b6] Dong X., You Y. & Wu J. Q. Building an RNA Sequencing Transcriptome of the Central Nervous System. Neuroscientist, doi: 10.1177/1073858415610541 (2015). PMC4833695 26463470

[b7] Edmondson R., Broglie J. J., Adcock A. F. & Yang L. Three-dimensional cell culture systems and their applications in drug discovery and cell-based biosensors. Assay Drug Dev Technol 12, 207–218, doi: 10.1089/adt.2014.573 (2014). 24831787 PMC4026212

[b8] Lammel S. . Unique Properties of Mesoprefrontal Neurons within a Dual Mesocorticolimbic Dopamine System. Neuron 57, doi: 10.1016/j.neuron.2008.01.022 (2007). 18341995

[b9] Brown A. L., Day T. A., Dayas C. V. & Smith D. W. Purity and enrichment of laser-microdissected midbrain dopamine neurons. Biomed Res Int 2013, 747938, doi: 10.1155/2013/747938 (2013). 23984404 PMC3741955

[b10] Arlotta P. . Neuronal subtype-specific genes that control corticospinal motor neuron development *in vivo*. Neuron 45, 207–221, doi: 10.1016/j.neuron.2004.12.036 (2005). 15664173

[b11] Apuschkin M. . A novel dopamine transporter transgenic mouse line for identification and purification of midbrain dopaminergic neurons reveals midbrain heterogeneity. The European journal of neuroscience 42, 2438–2454, doi: 10.1111/ejn.13046 (2015). 26286107 PMC4789538

[b12] Cahoy J. D. . A transcriptome database for astrocytes, neurons, and oligodendrocytes: a new resource for understanding brain development and function. J Neurosci 28, 264–278, doi: 10.1523/JNEUROSCI.4178-07.2008 (2008). 18171944 PMC6671143

[b13] Finegersh A. & Homanics G. E. Chromatin immunoprecipitation and gene expression analysis of neuronal subtypes after fluorescence activated cell sorting. J Neurosci Methods 263, 81–88, doi: 10.1016/j.jneumeth.2016.02.006 (2016). 26868730 PMC4801782

[b14] Wylot B., Konarzewska K., Bugajski L., Piwocka K. & Zawadzka M. Isolation of vascular endothelial cells from intact and injured murine brain cortex-technical issues and pitfalls in FACS analysis of the nervous tissue. Cytometry A 87, 908–920, doi: 10.1002/cyto.a.22677 (2015). 25892199

[b15] Yuan S. H. . Cell-surface marker signatures for the isolation of neural stem cells, glia and neurons derived from human pluripotent stem cells. PLoS One 6, e17540, doi: 10.1371/journal.pone.0017540 (2011). 21407814 PMC3047583

[b16] Russell J. N., Clements J. E. & Gama L. Quantitation of gene expression in formaldehyde-fixed and fluorescence-activated sorted cells. PLoS One 8, e73849, doi: 10.1371/journal.pone.0073849 (2013). 24023909 PMC3759445

[b17] Guez-Barber D. . FACS purification of immunolabeled cell types from adult rat brain. Journal of Neuroscience Methods 203, doi: 10.1016/j.jneumeth.2011.08.045 (2011). PMC3221768 21911005

[b18] Smith S. M., Kimyon R. S. & Watters J. J. Cell-type-specific Jumonji histone demethylase gene expression in the healthy rat CNS: detection by a novel flow cytometry method. ASN Neuro 6, 193–207, doi: 10.1042/AN20130050 (2014). 24735454 PMC4034710

[b19] Thavarajah R., Mudimbaimannar V., Elizabeth J., Rao U. & Ranganathan K. Chemical and physical basics of routine formaldehyde fixation. Journal of oral and maxillofacial pathology: JOMFP 16, 400–405, doi: 10.4103/0973-029x.102496 (2012). 23248474 PMC3519217

[b20] Shi S.-R., Key M. E. & Kalra K. L. Antigen retrival in formalin-fized, paraffin-embedded tissues: An enhancement method for immunohistochemical staining based on microwave oven heating of tissue sections. The Journal of Histochemistry and Cytochemistry: Official Journal of the Histochemistry Society 39, 741–748 (1991). 1709656 10.1177/39.6.1709656

[b21] Addis M. . Generation of high‐quality protein extracts from formalin‐fixed, paraffin-embedded tissues. PROTEOMICS 9, 3815–3823, doi: 10.1002/pmic.200800971 (2009). 19637237

[b22] Magdeldin S. & Yamamoto T. Toward deciphering proteomes of formalin‐fixed paraffin-embedded (FFPE) tissues. PROTEOMICS 12, 1045–1058, doi: 10.1002/pmic.201100550 (2012). 22318899 PMC3561704

[b23] Gustafsson O., Arentz G. & Hoffmann P. Proteomic developments in the analysis of formalin-fixed tissue. Biochimica et Biophysica Acta (BBA) - Proteins and Proteomics 1854, doi: 10.1016/j.bbapap.2014.10.003 (2014). 25315853

[b24] Ikeda K. . Extraction and analysis of diagnostically useful proteins from formalin-fixed, paraffin-embedded tissue sections. The Journal of Histochemistry and Cytochemistry: Official Journal of the Histochemistry Society 46, 397–403 (1998). 9487122 10.1177/002215549804600314

[b25] Dennis K., Uittenbogaard M., Chiaramello A. & Moody S. A. Cloning and characterization of the 5–flanking region of the rat neuron-specific Class III B-tubulin gene. Gene 294, 267–277 (2002). 10.1016/s0378-1119(02)00801-6 12234689

[b26] Kawashima Y., Kodera Y., Singh A., Matsumoto M. & Matsumoto H. Efficient extraction of proteins from formalin-fixed paraffin-embedded tissues requires higher concentration of tris(hydroxymethyl)aminomethane. Clinical Proteomics 11, 4, doi: 10.1186/1559-0275-11-4 (2014). 24484752 PMC3922997

[b27] Mahmood T. & Yang P. C. Western blot: technique, theory, and trouble shooting. N Am J Med Sci 4, 429–434, doi: 10.4103/1947-2714.100998 (2012). 23050259 PMC3456489

[b28] Shi S. R., Shi Y. & Taylor C. R. Antigen retrieval immunohistochemistry: review and future prospects in research and diagnosis over two decades. J Histochem Cytochem 59, 13–32, doi: 10.1369/jhc.2010.957191 (2011). 21339172 PMC3201121

[b29] Tashiro K., Hasegawa M., Ihara Y. & Iwatsubo T. Somatodendritic localization of phosphorylated tau in neonatal and adult rat cerebral cortex. NeuroReport 8, 2797–2801 (1997). 9295120 10.1097/00001756-199708180-00029

[b30] Burack M. A. & Halpain S. Site-specific regulation of alzheimer-like tau phosphorylation in living neurons. Neuroscience 72, 167–184 (1996). 8730715 10.1016/0306-4522(95)00546-3

[b31] Maragakis N. J. & Rothstein J. D. Mechanisms of Disease: astrocytes in neurodegenerative disease. Nat Clin Pract Neurol 2, 679–689, doi: 10.1038/ncpneuro0355 (2006). 17117171

[b32] Kahlson M. A. & Colodner K. J. Glial Tau Pathology in Tauopathies: Functional Consequences. J Exp Neurosci 9, 43–50, doi: 10.4137/JEN.S25515 (2015). 26884683 PMC4750898

[b33] Marion-Poll L., Montalban E., Munier A., Herve D. & Girault J. A. Fluorescence-activated sorting of fixed nuclei: a general method for studying nuclei from specific cell populations that preserves post-translational modifications. Eur J Neurosci 39, 1234–1244, doi: 10.1111/ejn.12506 (2014). 24713002

[b34] Gry M. . Correlations between RNA and protein expression profiles in 23 human cell lines. BMC Genomics 10, 365, doi: 10.1186/1471-2164-10-365 (2009). 19660143 PMC2728742

[b35] Vogel C. & Marcotte E. M. Insights into the regulation of protein abundance from proteomic and transcriptomic analyses. Nat Rev Genet 13, 227–232, doi: 10.1038/nrg3185 (2012). 22411467 PMC3654667

[b36] de Sousa Abreu R., Penalva L. O., Marcotte E. M. & Vogel C. Global signatures of protein and mRNA expression levels. Mol Biosyst 5, 1512–1526, doi: 10.1039/b908315d (2009). 20023718 PMC4089977

[b37] Bandeira F., Lent R. & Herculano-Houzel S. Changing numbers of neuronal and non-neuronal cells underlie postnatal brain growth in the rat. Proc Natl Acad Sci USA 106, 14108–14113, doi: 10.1073/pnas.0804650106 (2009). 19666520 PMC2729028

[b38] Darmanis S. . A survey of human brain transcriptome diversity at the single cell level. Proc Natl Acad Sci USA 112, 7285–7290, doi: 10.1073/pnas.1507125112 (2015). 26060301 PMC4466750

[b39] Sergeant N., Delacourte A. & Buee L. Tau protein as a differential biomarker of tauopathies. Biochim Biophys Acta 1739, 179–197, doi: 10.1016/j.bbadis.2004.06.020 (2005). 15615637

[b40] Iqbal K., Liu F. & Gong C. X. Tau and neurodegenerative disease: the story so far. Nat Rev Neurol 12, 15–27, doi: 10.1038/nrneurol.2015.225 (2016). 26635213

[b41] Kovacs G. G. Invited review: Neuropathology of tauopathies: principles and practice. Neuropathol Appl Neurobiol 41, 3–23, doi: 10.1111/nan.12208 (2015). 25495175

[b42] Buee L., Bussiere T., Buee-Scherrer V., Delacourte A. & Hof P. R. Tau protein isoforms, phosphorylation and role in neurodegenerative disorders. Brain Research Reviews 33, 95–130 (2000). 10967355 10.1016/s0165-0173(00)00019-9

[b43] LoPresti P., Szuchet S., Papasozomenos S. C., Zinkowski R. P. & Binder L. I. Functional implications for the microtubule-associated protein tau: Localization in oligodendrocytes. Proc Natl Acad Sci USA 92, 10369–10373 (1995). 7479786 10.1073/pnas.92.22.10369 PMC40798

[b44] Ferrer I. . Glial and neuronal tau pathology in tauopathies: Characterization of disease-specific phenotypes and tau pathology progression. J Neuropathol Exp Neurol 73, 81–97 (2014). 24335532 10.1097/NEN.0000000000000030

[b45] Das V., Sim D. A. & Miller J. H. Effect of taxoid and nontaxoid site microtubule-stabilizing agents on axonal transport of mitochondria in untransfected and ECFP-htau40-transfected rat cortical neurons in culture. J Neurosci Res 92, 1155–1166, doi: 10.1002/jnr.23394 (2014). 24788108

[b46] MacDonald C., Unsworth C. P. & Graham E. S. Enrichment of differentiated hNT neurons and subsequent analysis using flow-cytometry and xCELLigence sensing. J Neurosci Methods 227, 47–56, doi: 10.1016/j.jneumeth.2014.02.004 (2014). 24530700

[b47] Lein E. S. . Genome-wide atlas of gene expression in the adult mouse brain. Nature 445, 168–176, doi: 10.1038/nature05453 (2007). 17151600

[b48] Cheng L. . Laser-assisted microdissection in translational research: Theory, technical considerations, and future applications. Applied Immunohistochemistry & Molecular Morphology 21 (2013). 10.1097/PAI.0b013e31824d0519 22495368

[b49] Dingle Y. T. . Three-Dimensional Neural Spheroid Culture: An *in vitro* Model for Cortical Studies. Tissue Eng Part C Methods 21, 1274–1283, doi: 10.1089/ten.TEC.2015.0135 (2015). 26414693 PMC4663656

[b50] Jiang X. . Development of efficient protein extraction methods for shotgun proteome analysis of formalin-fixed tissues. Journal of proteome research 6, 1038–1047, doi: 10.1021/pr0605318 (2007). 17266348

