# Supplementary material for: Protein characterization of intracellular target-sorted, formalin-fixed cell subpopulations
Source: Sci Rep. 2016 Sep 26;6:33999. doi: 10.1038/srep33999 (PMC5036045; doi:10.1038/srep33999)
Supplement: Supplementary Information [file srep33999-s1.pdf]

Title: Protein characterization of intracellular target-sorted, formalin-fixed cell subpopulations

Full names:
Jessica S. Sadick[1]
Molly E. Boutin[1,2]
Diane Hoffman-Kim[1-4]
Eric M. Darling[1,2,4,5*]

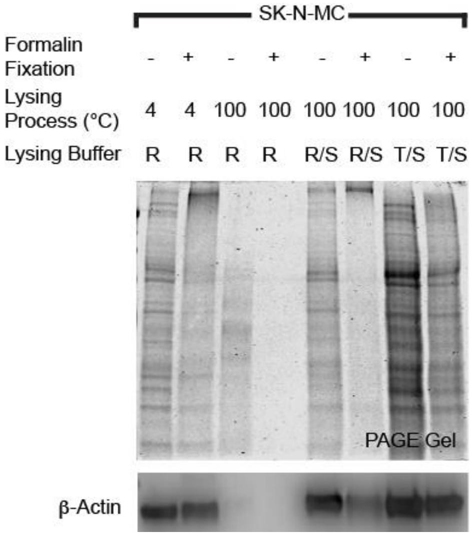

Supplementary Figure S1. **Lysis protocol evaluation.** Trial run assessing lysis buffers ability to extract protein from fresh and formalin-fixed samples as compared by Coomassie Blue stained PAGE gels and WB. R, radioimmunoprecipitation assay (RIPA) buffer; R/S, RIPA buffer and 2% SDS; T/S, 300 mM Tris-HCl and 2% SDS. All lysis buffers had 2X protease and phosphatase inhibitor cocktail.

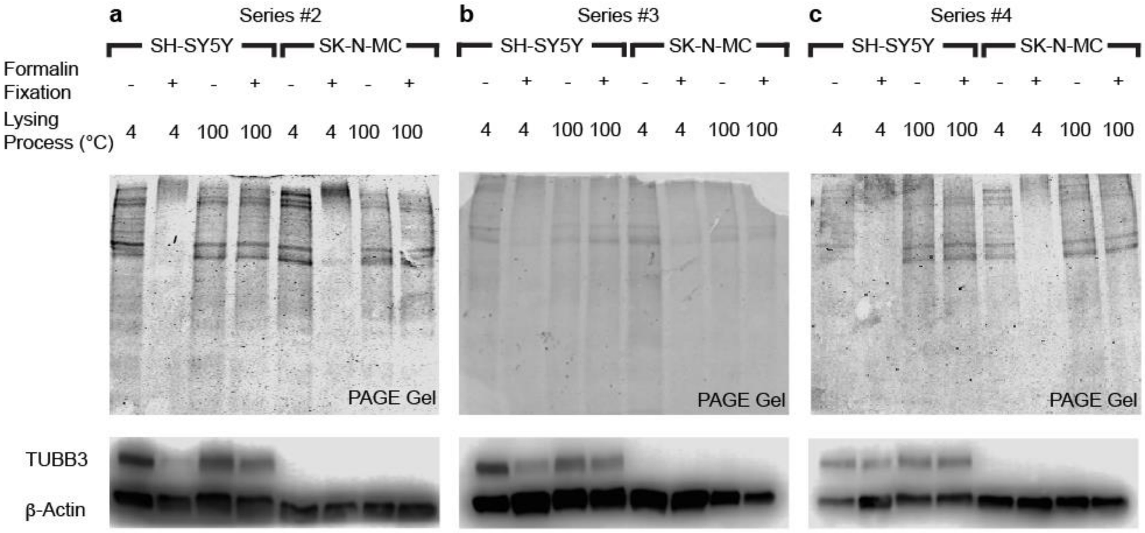

Supplementary Figure S2. **Protein extraction and lysis protocol independent replicates.** (a) Series 2, (b) Series 3, and (c) Series 4 validating protein extraction from fresh and formalin-fixed samples as compared by Coomassie Blue stained PAGE gels and WB. Series 1 is presented in Fig. 1.

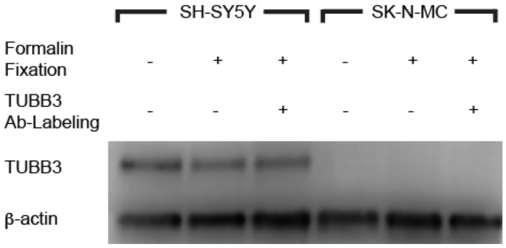

Supplementary Figure S3. **TUBB3 antibody-labeling of cells did not interfere with subsequent WB probing for the same epitope.**

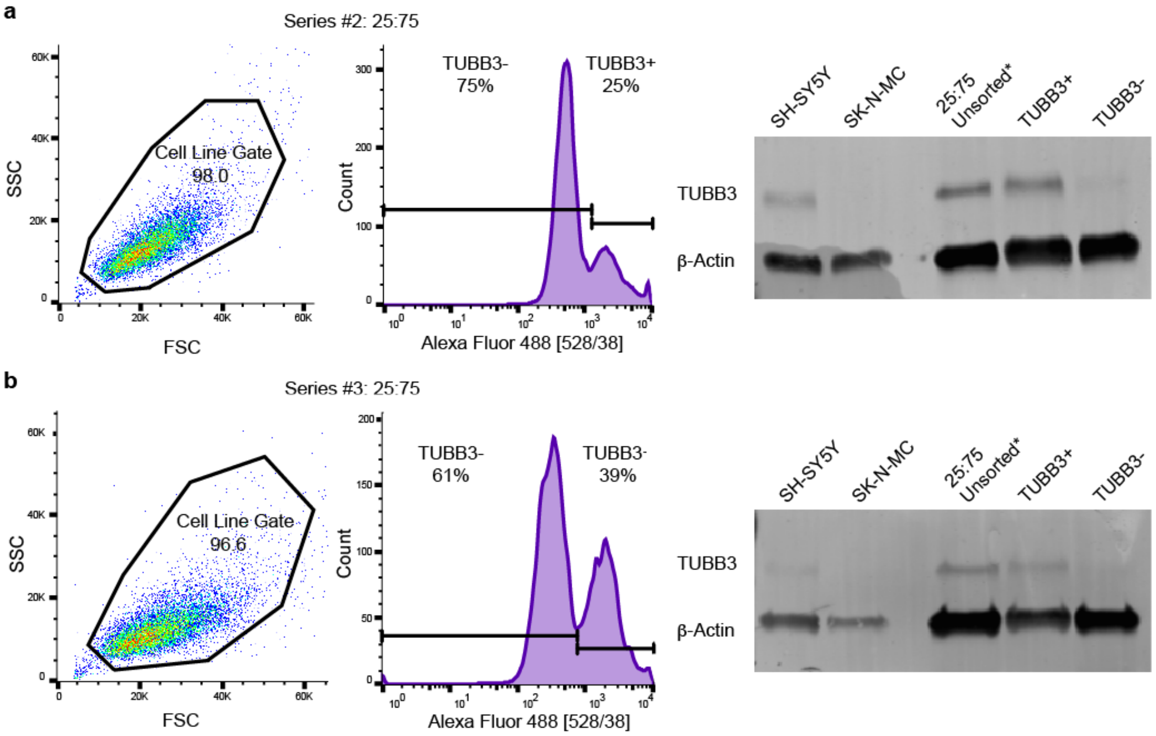

Supplementary Figure S4. **Cell line, controlled-ratio independent replicates.** (a) Series 2 and (b) Series 3 of intracellular target-based FACS validation experiments using artificially heterogeneous cell line populations. 25:75 unsorted* samples were not run through the sorter. Series 1 is presented in Fig. 2.
